# Mind the Gap: A Qualitative Study of Patients’ Experiences of Acute Elective Care Across the NHS and the Private Sector in England

**DOI:** 10.1111/hex.70867

**Published:** 2026-09-15

**Authors:** Eleanor Gee, Gemma Stringer, Jane Ferguson, Michael Molete, Kieran Walshe, Christos Grigoroglou, Thomas Allen, Michael Anderson, Nils Gutacker, Karen Bloor

**Affiliations:** ^1^ Alliance Manchester Business School The University of Manchester Manchester UK; ^2^ Division of Population Health, Health Services Research & Primary Care University of Manchester Manchester UK; ^3^ Health Services Management Centre University of Birmingham Birmingham UK; ^4^ Manchester Centre for Health Economics, Division of Population Health, Health Services Research and Primary Care The University of Manchester Manchester UK; ^5^ Health Organisation, Policy, and Economics (HOPE), Division of Population Health, Health Services Research and Primary Care University of Manchester Manchester UK; ^6^ Centre for Health Economics University of York York UK; ^7^ Department of Health Sciences University of York York UK

**Keywords:** NHS, patient choice, patient experience, patient safety, private hospitals, qualitative

## Abstract

**Introduction:**

Whilst the use of the private sector to deliver acute elective care in the United Kingdom is increasing, there is limited understanding of the patient experience when moving between NHS and private organisations to receive treatment. This study aims to explore how patients make choices and navigate care pathways across NHS and private sector organisations.

**Methods:**

A qualitative semi‐structured interview study was conducted with 27 patients and 73 clinical and non‐clinical senior leaders across NHS and private organisations between September 2023 and April 2025. Analysis was conducted using a reflexive thematic approach and abductive analysis to position themes against wider knowledge.

**Results:**

Three key themes structured around the patient journey were identified through analysis. First, the ability of patients to make informed decisions on receiving treatment in private sector organisations was constrained by long waiting times and challenges accessing NHS care. Second, there was a lack of consistency in the timely and thorough transfer of patient information between NHS and private sector organisations, which undermined patient trust in the safe provision of care when delivered across the interface. Third, the provision of aftercare across the interface was fragmented, and its effects compounded by ambiguity for patients, and at times, providers, for who was responsible for the ongoing management of patient care once they had received treatment in a private hospital.

**Conclusion:**

Patients receiving elective care across the NHS and the private sector faced some barriers in decision‐making, transfer of information and aftercare. In order to address these challenges, there is a need for stronger collaboration between the sectors to improve information sharing, continuity of care and patient safety across the interface.

**Patient or Public Contribution:**

Patients and carers were involved in our study from inception to dissemination. Our Patient and Public Involvement (PPI) forum was involved throughout project design and planning and gave us feedback and guidance on research materials and outputs (e.g., interview schedules, participant recruitment material, theme development). Our PPI group co‐produced our patient interview schedule and were involved in analysis of patient interviews. Our PPI co‐applicant was involved in the preparation of this manuscript.

## 1 Introduction and Background

Patients in England access private sector healthcare for a range of clinical needs, funded by NHS referral, self‐payment or private insurance. Recent data shows that the admission of both NHS‐funded and self‐funded patients in private sector organisations in England has been steadily increasing, with the proportion of NHS funded elective admissions to private sector organisations rising from 5.6% in 2019/2020 to 7.5% in 2022/2023 [1] and the proportion of all privately funded elective admissions growing from 7.4% in 2019/2020 to 8.3% in 2022/2023 [2]. In January 2025, the UK government announced an agreement to further expand the use of the private sector for the provision of NHS‐funded care to cut growing waiting lists [3]. Such developments not only reflect systemic changes in healthcare delivery but may also impact patient choice, autonomy and experience in an increasingly hybrid healthcare landscape. Internationally, many healthcare systems have similarly complex combinations of public and private healthcare provision, and concerns about differential performance, access and care transitions are often noted [4, 5]. Research from countries with mixed private and public models of care such as Australia and Sweden shows challenges, including limited information to support patient choice, barriers to access and a shift in responsibility when choosing appropriate healthcare services from the healthcare systems onto the individual patients [6, 7].

The motivations behind increased use of the private sector to deliver patient care in England are often framed as driving greater efficiency of healthcare provision and enhanced patient choice [8]. Given that record‐high public dissatisfaction with the NHS is closely linked to difficulties in receiving timely care [9], improving access to and experience of healthcare is significant not just for improving health outcomes for patients but for rebuilding public confidence in the capacity of government to deliver effective services. The increased role of the private sector inevitably adds complexity to the patient experience of care, from patient choice on where to be treated, to how care is managed between sectors, yet there is limited understanding of the patient experience of care when moving between NHS and private organisations. As the role of the private sector in delivering NHS and private care continues to grow, attention must turn to how patients navigate choices, and what their experiences reveal about quality and safety across the interface of public and private healthcare.

In recent decades, there have been cross‐organisational efforts to strengthen informed patient choice. The NHS constitution pledges to provide patients with ‘*easily accessible, reliable and relevant information… to participate fully in your own healthcare decisions and to support you in making choices’* [10]. From 2004, the Department for Health introduced ‘Choose and Book’, later replaced by the NHS e‐Referral Service, which provides patients the opportunity to choose any provider with an NHS contract to deliver their care once referred for a consultant‐led treatment, with a small number of exceptions [11, 12]. In 2014, the Competition and Markets Authority mandated the collection and publication of consultant data by the Private Hospital Information Network (PHIN) to provide patients with data on safety and clinical outcomes of private healthcare providers across the United Kingdom to make informed decisions when receiving care in private hospitals [13]. National Institute for Health and Care Excellence (NICE) guidelines also provide recommendations for providers to support patients in making holistically informed decisions on treatment most suited to their personal preferences and priorities [14]. However, despite these efforts, evidence suggests that the absence of clear, accessible information on consultants’ private practice activity, and support available to help patients engage with this information, undermines informed decision‐making [15, 16, 17].

This is contextualised by recent findings highlighting that patient awareness of private healthcare in the United Kingdom is still limited, with 43% of respondents reporting that they are not very, or not at all familiar with how to access private healthcare [18]. The lack of awareness of accessible information to support patient decision‐making, coupled with the lack of familiarity in navigating private healthcare in the United Kingdom, means that patient decision‐making is often not based on information regarding safety and clinical outcomes. Rather, patient choice literature often finds that patients primarily make decisions on where to be treated based on the reputation and proximity of the hospital, waiting times, and cost of treatment [8, 18, 19, 20].

Haggerty et al. describes three types of continuity of care: informational, management, and relational, which relate to the availability of complete patient information, coordinated processes across settings, and ongoing trust in providers [21]. Considering these dimensions provides a useful framework for understanding why transitions between services may disrupt patients’ experiences of continuous care [21]. Though there is limited empirical literature on the patient experience of care across the interface of public and private organisations, research has found that transition points between same‐sector care settings and services undermine continuity of care for patients, partly due to the challenges of sharing information, negatively impacting patient's clinical outcomes and their confidence in healthcare professionals [22, 23]. This has also been found to be the case within organisations, where fragmented transitions between inpatient and outpatient care are impeded by incomplete or unavailable patient records, at times necessitating the patient to manage the transfer of their own records [24]. Research on the patient experience of aftercare has also highlighted shortfalls in communication when patients access care across multiple NHS organisations, adversely impacting the quality of aftercare that they received [25].

To date, there is limited understanding of the patient experiences of care when moving across the interface of NHS and private healthcare organisations. The aim of this research was to explore the patient experience across the elective care pathway between NHS and private sector hospitals in England, to identify opportunities to improve the delivery of safe, high‐quality care.

## 2 Methodology

### 2.1 Study Design and Context

Qualitative interviews were conducted with patients and clinical and non‐clinical senior leaders with clinical governance responsibilities across NHS and private sector hospitals and organisations in England. A semi‑structured interview schedule was developed and reviewed by the project's PPI forum. It explored patient experiences of accessing NHS and private care, their choice of private hospital and their treatment, safety, and aftercare. Topics included waiting times, funding, information provided, staff knowledge, any complications, and the overall impact of care. This paper predominantly draws on findings from one‐to‐one interviews with patients. However, we have also drawn on findings from the wider data set of interviews with clinical and non‐clinical senior leaders in NHS and private healthcare organisations in England for a comprehensive analysis of the patient experience of care across the interface. The study received ethical approval from the Health Research Authority (ethical approval number removed for anonymity). All participants provided written informed consent.

### 2.2 Recruitment

Clinical and non‐clinical senior leaders were identified through a combination of purposive and snowball sampling and included individuals working in either an NHS (*n* = 34) or private healthcare organisation (*n* = 27), both (*n* = 11) or in a professional membership organisation (*n* = 1), such as chief medical officers, hospital directors, responsible officers and clinical governance leads. Patient and carer participants were recruited through patient support groups and charities (*n* = 18), social media groups (*n* = 4), National Institute for Health and Care Research (NIHR) recruitment initiative Research for the Future (*n* = 4), word of mouth (*n* = 3) and through an NHS case study site (*n* = 1). Patient and carer participants included individuals from both funding types (NHS or self‐funded, either through upfront payment or private medical insurance) who had received care for the same acute health condition across both sectors. Specifically, patients had accessed healthcare services in the NHS and had also received treatment for that condition in a private hospital. Acute health conditions included, but were not limited to, hip replacements, cataracts and organ transplants. Individuals were excluded if they had experience of only one sector (NHS or private), or if they had used NHS and private services for different health conditions.

Individuals expressed interest via email and were subsequently invited to a screening call. One researcher (EG) conducted a short screening phone call with all individuals who expressed interest in participating in patient and carer interviews to provide an overview of the project and the reasons for doing the research and to ensure that the inclusion criteria were met. During this process, it was suspected that several imposter participants had applied to participate based on the format of some enquiries that were received. The term imposter participants refers to individuals who apply to participate in research but misrepresent themselves or their experiences [26, 27, 28]. As a result, screening call questions were added to verify eligibility and notes were taken of the screening process with impressions of participants honesty (see Supporting Information S1 for further details) [29]. Notes were reviewed collectively by the research team, enabling decisions to be made, discussed, corroborated and documented.

Participants received a £25 Amazon voucher or bank transfer payment in recognition of their time and contribution to the study.

### 2.3 Data Collection

The schedule for interviews with clinical and non‐clinical senior leaders was informed by a review of relevant policy documents and preliminary discussions with key leaders in the NHS and private sector to explore clinical governance arrangements between NHS and private healthcare organisations (see Supporting Information S1 for further details) [29]. Notes were reviewed collectively by the research team (Supporting Information S3). The schedule for interviews with patients was designed by the research team in collaboration with our patient and public involvement (PPI) forum and was informed by a review of existing literature on clinical governance, patient safety and patient choice (see Supporting Information S2). Both sets of interview schedules evolved iteratively as interviews progressed. Five authors (E.G., G.S., J.F., M.M. and K.W.) conducted 27 interviews with patients, and 73 interviews with clinical and non‐clinical senior leaders in NHS and private healthcare organisations. No participants dropped out or refused to participate. Data were collected face to face and via video conferencing software, with just the participant and researcher present. Table 1 presents the characteristics of participants in the study. Data collection occurred between September 2023 and April 2025, interviews lasted 48 min on average (range = 15–108 min) and audio recordings of the interviews were transcribed verbatim. The research team met at regular intervals to discuss the progress of interviews and completed data collection once it was agreed that theoretical saturation had been reached [30].

**Table 1:** Characteristics of study participants.

Characteristic			Patients	Senior leaders
Mean age (SD)^a^			60 (15)^a^	52 (10)^a^
Gender		Women	17 (63)	31 (43)
		Men	9 (33)	42 (58)
		Transgender women	1 (4)	0
Ethnicity	White	English/Welsh/Scottish/Northern Irish/British	21 (78)	51 (76)
		Any other White background	1 (4)	5 (8)
	Mixed/Multiple ethnic groups	White Asian	0	1 (2)
	Asian/Asian British	Indian	1 (4)	6 (9)
		Pakistani	2 (8)	1 (2)
		Bangladeshi	1 (2)	0
	Black/African/Caribbean/Black British	African	1 (4)	1 (2)
	Other ethnic group	Arab	0	1 (2)
		Persian	0	1 (2)

*Note:* Data are presented as frequency (%) unless otherwise indicated.

^a^ SD. Not all senior leader participants provided age and ethnicity information.

### 2.4 Analysis

The NVivo software package was used to organise the data into codes and themes through the adoption of reflexive thematic analysis (RTA) [31]. RTA involved familiarisation with the data through repeated readings of the transcripts; generation of initial codes from which broader themes were developed; to support this process, we compared our interpretations and discussed different ways of grouping the coded material into coherent candidate themes; these themes formed the foundation for patterns of meaning throughout the dataset; the revision and definition of the key themes from the data, and the analytical write‐up to situate the findings within the relevant literature [31]. Analysis was conducted by five researchers (E.G., G.S., J.F., M.M. and K.W.), two male and three female. Regular meetings were held to discuss initial findings and theme development, and to engage in critical reflexive dialogue to examine assumptions and interpretations. The research team included academic researchers from universities in England with doctoral qualifications and expertise in qualitative health research, alongside a PPI co‐investigator who is a patient champion, carer ambassador, and qualified nurse not currently employed by the NHS. All team members have personal experience of NHS‐funded privately provided care, and these perspectives were acknowledged and reflected upon throughout the analytic process. Key themes were also shared and discussed with the project advisory group and PPI forum throughout the data collection period to further scrutinise theme development and offer alternative interpretations for enhanced rigour.

## 3 Results

The results section of this paper is structured around three key stages of the patient journey identified through analysis: patient choice, administrative transitions and aftercare. Within each stage, several sub‑themes illustrate how patients experience the NHS–private sector interface and how these interactions affect their experience of quality and safety. The section begins by examining what influences patient choice, including patients’ understanding of the two sectors, the availability of information, the impact of long NHS waits and the role of familiarity and recommendations. It then explores administrative transitions, focusing on the sharing of health records and the continuity of information across providers. Finally, it considers aftercare, including perceptions of responsibility for follow‑up care and how patients respond when complications arise.

### 3.1 Theme 1: Patient Choice

#### 3.1.1 Patient Understanding of the Sectors and Information Availability

When making decisions about whether to be treated in an NHS or private hospital, patients’ knowledge about the hospital, consultant and potential benefits and risks associated with their treatment varied considerably. While some senior leaders thought that certain patients already had a clear understanding of the differences between NHS and private hospitals, particularly regarding the absence of critical care support and the protocols for managing complications, others encountered patients for whom the private sector was an ‘enigma’ that was unfamiliar or difficult to understand. This lack of familiarity contributed to differing expectations of the nature and quality of care across the two settings.

‘I don't think they fully understand. And I think it varies between patient[s], as well. There's no set theme or trend with patients, with regards to their understanding of private sector hospitals… Their expectations, some are really, really grateful, some expect a lot more, if they've had an issue, will say, oh, that's because I'm an NHS patient. … they struggle to understand fully, their own care pathways sometimes, let alone the difference between the independent sector and the NHS’.

(Clinical Governance and Quality Manager, private sector)

‘I: Do you think patients themselves are aware of the differences… between being treated in an independent hospital versus being treated at [NHS hospital]?’

‘R: I don't think they are. I don't think they're given that level of detail about what is available in the hospital in terms of equipment’.

(Consultant Orthopaedic Surgeon, both NHS and private sector)

When faced with the decision to receive care in either an NHS or private hospital, and often in the absence of a clear understanding of how care is managed in each sector, a small number of patients we spoke to were proactive in seeking out information to guide their choice. This included searching online for regulatory reports, patient reviews and consultant profiles.

‘I started with just doing research on… the private hospital's website. There was probably about six or seven different gynaecologists that practised there. So I went through and read each person's description and what their clinical interests were, to see which person would align best with the treatment that I needed’.

(Patient interview 7, self‐funded)

Patients reported that there was not always a lot of information available online about consultants and it was often much easier to find information about a consultant's private practice than their NHS work, with consultants having online profiles on private hospital websites, and in some cases, their own websites detailing their clinical specialties and experiences.

‘If you go on [consultant's private practice website], you'll see all the sort of information on there, and then if you search him on the [private hospital website] or something, you'll see he's got a profile on there. And then you'll see all the information that he's got in abundance on his website, so as a patient I can go on there and see anything, you know, and all the information is on there. And if I'm a patient for the NHS, I've got nowhere to go, absolutely nowhere, I can't find any information, …You can't find anything about the surgeon, anything about your consultant, anything about your procedure, you don't know who you're seeing or… but yet privately, all that information is there’.

(Patient interview 13, self‐funded)

‘There isn't always a lot of data on them, I have to say, not enough to make a properly informed decision, I think it's fairly superficial’.

(Patient interview 6, NHS and self‐funded)

Of those patients who did seek out information on the consultant, hospital or procedure, none reported using data available online from PHIN, despite the intention of the organisation to support patients in making informed decisions when accessing private healthcare. Instead, the information sought was largely focused on the consultant's profile, rather than their clinical outcomes and safety record. More often though, patients did not report seeking out further information about the hospital, consultant or procedure before making their decision of where to be treated and did not report that they were encouraged by their NHS doctor to seek out this information when discussing their treatment options.

#### 3.1.2 Long Waiting Times and Difficulty Accessing NHS Care

Most patients cited long waiting times and difficulty accessing NHS care as the primary reason for choosing treatment in a private hospital, whether NHS‐funded or self‐funded. NHS‐funded patients often reported accepting referrals to private hospitals with limited information about the hospital or consultant, viewing it as a preferable alternative to enduring longer wait times and a deterioration of their condition while waiting to be seen by an NHS clinician. Some patients felt they were not offered an NHS care option. Because NHS waiting times were so long, they perceived that the private hospital was effectively presented as the only choice. Similarly, self‐funded patients cited barriers to accessing NHS care, such as difficulties getting appointments or experiencing long delays between appointments, as a key reason to pay privately for their treatment.

‘What we were told was that they didn't have the capacity because there were too many…the queue was so long and my mother is 93 and should be seen earlier, should be done earlier. And she was told, you would go to the private hospital and the surgery would be done there, that was what we were told, and I didn't see any reason to think there was something else’.

(Patient interview 9, NHS and self‐funded)

‘I was advised by the NHS consultant, well we understand the last time when you had a similar problem you went and did it privately. Could you do it again? I said well…I did then ask, what are the alternatives? The alternative was that, yes, it can be done…. But I would probably have to wait for it a couple of years, for the biopsy. And they advised me, you shouldn't wait that long’.

‘*So again, I didn't have any choice and it wasn't that anyone was helping me, where to go and whom to actually approach, it was up to me whom I was going to, so I rang the private hospital and I asked who can do it as early as possible?’* (Patient interview 10, self‐funded). Respondents from the private sector felt that patients were often grateful for the option of NHS‐funded treatment in a private setting, particularly when they were in pain and had experienced long waits for NHS care. They also noted that this sense of relief could be reinforced by the more comfortable environment offered in private hospitals:

‘To be honest, when I've spoken to patients here, they're usually quite grateful…we do a lot of hips and knees and they've been on the waiting list quite a long time and they're in pain… they feel quite privileged to come to [hospital name] and have their operation, you know, in a private room and probably a nicer environment than they might have had in the NHS’.

(Clinical lead, private sector)

Long waiting times adversely impacted patients’ ability and motivation to engage with information that would better inform their decision‐making, particularly on aspects of safety. One patient actively avoided asking for more information on the private hospital, consultant and procedure when making their decision of where to be treated, so that they would not be deterred from getting treated as quickly as possible.

‘No, and I didn't ask. And that was more because if anything had gone wrong with my heart, I'm assuming they'd have blue‐lighted me to [NHS hospital]. But if you voice it, you think about it, and if I'd have thought about it too long, I wouldn't have gone through with it’.

(Patient interview 14, NHS‐funded)

The notion that waiting times could override patients’ interest in safety information when making choices of where to be treated was corroborated in interviews with senior leaders. It was acknowledged that whilst there was variation in what patients knew and wanted to know when deciding where to be treated and by whom, many were ultimately unable to make informed decisions as they were constrained by long waiting times and deteriorating health conditions.

*I think things have changed dramatically since COVID. Pre COVID my private practice was driven almost fully by word of mouth. So what do patients know? …*

‘I think the disparity between supply and demand has become so critical that patients just want to have their operation done… It's changed since COVID and people are perhaps less discerning than they might have been pre COVID because actually if they're faced with a two year wait for a hip replacement and they're in agony it's well, actually, I just want this done’.

(Clinical Lead and Surgeon, NHS)

#### 3.1.3 Familiarity: Geography, Proximity and Recommendations

Patients generally described their decision‐making about which private hospital to use as occurring within a very limited context, often selecting from a small number of providers. NHS‐funded patients often recalled having limited or no choice on which private hospital to be treated in, largely due to the limited availability of private providers in the area. If offered a choice of private hospital, or in the case of patients that were self‐funding treatment, decisions were often based on familiarity with the hospital's location.

NHS‐funded patients often recalled making decisions about where to be treated during the same appointment in which they were informed of their options. As a result, their choices were largely limited to the information they already had, typically the hospital's location and its proximity. Given the pressures of long waiting times and difficulties in accessing NHS appointments promptly, patients were generally not encouraged to take additional time to seek further information. Doing so risked delaying their treatment and potentially worsening their health condition. For most patients who self‐funded their care, the primary motivation was to receive treatment quickly. Consequently, extending the time to receive treatment by seeking out new information was often seen as counterintuitive. Selecting a provider based on familiarity with its location offered enough assurance that their care would be delivered quickly and conveniently.

‘I: And what information were you provided with when you made your choice of hospital?’

‘R: I don't think I was provided with any. In fact, to be fair, looking back, it was quite bizarre because he just said to me in the appointment, right you can go to [private hospital] or [private hospital], which would you prefer. I didn't know whether one was better than the other or anything, I just knew one was in [UK city] and one was in [UK town] and it was easier to get to [UK town]’.

(Patient interview 22, NHS‐funded)

‘I: … And what did you know about the hospital before you went?’

‘R: Well, that's a difficult one to answer, I used to work in the [NHS hospital], so I already knew of the existence of the [private hospital]. I knew what services they offered. So when my GP referred me, I just accepted it without thinking’.

(Patient interview 2, NHS‐funded)

Some patients also reported that recommendations from friends, family and NHS doctors guided their decision of which private hospital to be treated in and by whom. Patients who received NHS‐funded treatment in a private hospital generally followed the referral by the NHS doctor, as it was often the quickest route to timely care. Knowing that family or people they knew had good experiences in the hospital where they were to be treated eased their minds on this.

‘Well, my granddaughter had had minor surgery there for a problem she had…she was looked after really well and it was very quick… And, you know, various people that I've spoken to were…you know, I haven't heard a bad word about it, so…whereas you know, people have sort of said about… Our main hospitals … But, you know, when I sort of walk through the hospital, you know, it's just so untidy and it's just not very welcoming really. No, that's the only experience I've got really of the big hospitals. Oh and my daughter in law, she's had a few operations … she really didn't have very good care at all, in a big hospital’.

(Patient interview 15, NHS funded)

The significance of recommendations was stronger for self‐funded patients who had more capacity to consult friends, family and their NHS doctor before making their decision of where to be treated. Patients described how knowing someone who had received good quality care in a particular private hospital provided sufficient reassurance about the level of care they could expect. Some patients also reported selecting a particular private hospital based on conversations with their NHS doctor, who recommended colleagues practicing in nearby private hospitals. From this, patients felt sufficiently confident to select that clinician and hospital for their own treatment.

‘I had medical insurance. So, there wasn't really a strong thought process. I needed orthopaedic surgery. I asked a chap who I know very well and he looks after the pensions for footballers and sportsmen. And I said, where would they go, you know? And he said, oh, that's easy, they go and see Mr such‐a‐body. So, I went to see Mr such‐a‐body’.

(Patient interview 9, self‐funded)

‘I think they trust the NHS, and I think they know that most of the doctors that work in the private sector, come from the NHS, so I think that's where they get their trust level from. Beyond that, I don't know how they get their assurance around the safety and the quality that they get when they do have their care, because at the moment people are so obsessed with waiting times and getting seen quicker and getting a diagnosis and getting a decision, I think that over‐rides any level of quality of care that you might receive within a different organisation’.

(Chief Nurse, NHS)

### 3.2 Administrative Transitions

#### 3.2.1 Sharing Health Records Across the Sectors

There was variation across patient accounts on how their healthcare records were managed and transferred between sectors. Some patients reported that their records were passed between the two sectors adequately, particularly if they were on an NHS‐funded care pathway. In some instances, familiarity between organisations and/or clinicians supported administrative transitions across the interface, with clinicians contacting their NHS or private sector counterpart directly to discuss the patients’ treatment history.

‘When I got into the NHS, they were asking me what the story was. And I obviously told them, X weeks ago I had this operation at the [private hospital]. Oh, yes, who did it? Mr Such‐a‐body. Oh, yeah, we know him, I'll have a word with him, kind of thing. So, they then rang him and they came back to me and they said, yeah, I've spoken to [the consultant] and he said he's done this and this and this. So, we know where you're up to and we know what was done’.

(Patient interview 20, both NHS and self‐funded)

‘She had a full letter… So she knew everything… the letter was two pages long. It wasn't, oh, this lady's had a hip replacement. It was what they'd done, even down to the measurements of the prosthetic. Yeah, it was a very detailed letter. I've got a copy and obviously the GP had a copy’.

(Patient interview 4, NHS‐funded)

Some participants reported that they needed to repeat information, particularly within the NHS, but they understood why this might be necessary when referrals may have happened in the past, and health may have changed in the meantime.

‘I mean, they should have all this information the GP provided accessible, but they were asking so many questions. And I don't see there is any harm, but I just feel like it's repeating the same information, which they shouldn't have to do. But I was happy with that, because GP has sent the information probably six months ago, and in the meantime many things may have changed… The private one, they already knew all the information, so they were more up to date. Whereas NHS, they referred her one year and a half ago, so they had to, I think, reasonably adjust and verify all this information’.

(Patient interview 3, NHS‐funded)

However, more often, patients reported that their healthcare records were incomplete, lacked detail or were delayed in transference both between NHS organisations and across the interface. Some patients managed the transfer of their own records, citing previous negative experiences or concerns that the organisations would not do so accurately or in sufficient detail.

‘The main problem as far as the private hospital's concerned was of course the inability to transfer records and data. I've always found that the only way that I can control my patient record anyway is to manage it myself… I go on to all the sites, I collect all the test results, I cut and paste them, I put them together and I send them to the [consultant] or the [other consultant] so that they share. And also to the GP. Because none of my patient records are satisfactory on their own. I wouldn't want to be treated in any of them, in a particular trust, because they're never complete. So, I mean, there is obviously a major problem in transferring information. Every time I go into a private hospital, we start from scratch. Any records they've got are the records which they've got in that particular private hospital’.

(Patient interview 4, self‐funded)

‘With the NHS the notes… they weren't there on time. So we had to chase the notes up as well… When I was under the NHS the part of the day was he hadn't had the results back for a blood test and other things from my GP, and I just…you should have had them by now, he expected them. He was apologetic. But that wasn't good enough because I'm in all this pain and what I'm going through I thought oh no, we're not getting anywhere with this’.

(Patient interview 18, NHS and self‐funded)

There were organisational efforts to improve information sharing between the sectors. One private sector governance lead described best practices for sharing health records, explaining how their organisation sought to minimise handover points in order to reduce errors and improve patient experience:

‘…we all know the point at which you hand over care, there's risk, so the fewer points of handover that you can have, I think the better generally for the patient, and continuity of care, otherwise patients have to jump around an awful lot. And if you're thinking about that from a patient experience point of view, again, that's not great’.

(Governance lead, private sector)

#### 3.2.2 Informational Continuity

The reliance on the patient's own knowledge of their condition and treatment undermined patient safety and had a detrimental effect on the wellbeing of some of the patients we spoke to. Some patients expressed frustration that they were required to recall their own health history and questioned the safety of the need to do so. This concern was particularly felt when patients were recovering from their procedure. The absence of detailed information of their treatment and condition at a time when patients were particularly vulnerable highlighted the risks that the mismanagement of patient records across the interface poses to patient safety.

‘There was no communication back from that [private hospital] at all to the NHS. The only common link was me as the patient and the surgeon. There was nothing else. So, as I say, there was no information about the prescription, nothing carried across to my record. When I went back to the NHS for that first follow‐up, the records… weren't there of what I'd had done. So, it was only me telling the nurse that I'd had half my lung removed. That's not safe care… So no, the communication was really weak and poor’.

(Patient interview 23, NHS‐funded)

### 3.3 Aftercare: Responsibility Across the Interface

#### 3.3.1 Responsibility for Aftercare

Patients largely described their experiences of care in the private hospital as positive, recalling that the environment was cleaner, calmer, and staff were more attentive and efficient compared to care in the NHS. However, a point of vulnerability for patients often occurred in the period following their procedure, particularly if they experienced complications. There was a lack of clarity as to who was responsible for aftercare, particularly when NHS patients were treated in private hospitals. For self‐funded patients, there was an assumption that aftercare would be fully covered by the private hospital as part of their package of treatment. However, some were unaware that the private hospital may not have had the clinical capabilities to manage this, particularly if the patient experienced complications. Lack of clarity about who was responsible for aftercare led to inconsistencies with the quality of information, resources and the support patients received across both sectors as well as difficulties accessing follow‐up treatment or any remedial treatment.

‘The [private hospital] were saying well… now you've got this infection at this stage, it's not our responsibility, get back in touch with your GP. Then the GP was like well hang on, why have you had it done privately, what's that, it's not my responsibility. So it was almost like it's the end, I think, it's got to be more succinct and that aftercare is really, really key. Because when I've had surgery or any treatment through my private medical insurance or anything like that, it's that aftercare it's seamless. You kind of feel really comfortable and confident that somebody's taking the best care of you as possible. Whereas I think with that clunkiness of that NHS, private, the NHS, it was almost like nobody was taking accountability or responsibility for it’.

(Patient interview 24, NHS‐funded)

#### 3.3.2 When Things Go Wrong: Understanding and Responsibility

There were also differing opinions on who should be responsible for their care when things went wrong, especially when patients were self‐funded. NHS‐funded patients generally felt that following their procedure in the private hospital, responsibility for aftercare transferred back to the NHS. However, some self‐funded patients felt that the private hospital should have taken more responsibility if there was a complication in their recovery as they had paid for the treatment. Some patients expressed frustration at having been directed back to the NHS for remedial treatment for complications developed from their surgery in the private hospital. The ambiguity with regard to who was responsible for their care once they had developed complications demonstrated that patients were not always well informed of the arrangements across their full care pathway, prior to their treatment.

‘Obviously, I left the hospital fine, I had, you know, stitches but then there was a problem. And it's interesting because looking on the hospital website, there's no real mention about aftercare or…obviously, it tells you how to make a complaint but there's no real mention of if things go wrong, you know, what do you do in this situation?’

‘Because sometimes with an operation…I mean, I know these things happen in terms of infections. I think the problem I had was, I just didn't feel it was being taken seriously. And as I say, contacting the secretary when I started feeling unwell and getting a response, go to A&E, just didn't wash with me. You see what I mean, you expect the surgeon to really take responsibility’.

(Patient interview 5, NHS funded)

The notion that patients were not always informed of the differences between sectors, particularly when complications arose, was echoed in interviews with senior leaders. It was felt that when patients lack a clear understanding of differences between NHS and private hospitals, and how their care is managed between them, it can heighten patients’ confusion and frustration if their treatment deviates from expectations.

‘So when things go wrong in the private sector and patients get transferred into the NHS for treatment, in my experience, there's a very high volume of at least complaint and not infrequently litigation, some of it justified, some of it perhaps not justified, because anyone can have a bad day in the operating theatre… particularly patients, in my experience, who are fee paying private patients, fail to understand that just because they're going somewhere shiny, with nice carpets, and having prawn sandwiches, you know, that doesn't mean that their operation won't go wrong and they won't have to be transferred somewhere else to have it sorted out. But I think the gap between expectation and outcome is bigger in those cases and it's certainly associated with considerably more dissatisfaction, if not litigation’.

(Consultant Surgeon, NHS)

## 4 Discussion

This research has highlighted the complex nature of the patient journey in elective care across NHS and private hospitals. Although patients particularly valued the reduced waiting times for care that were offered by private hospital, they sometimes encountered challenges that compromised the quality and safety of their care at transition points between sectors, both prior to and following treatment, from the selection of treatment provision to the coordination of care across organisations. Despite policy directives aimed at engaging patients to make fully informed choices on their treatment, our findings indicate that patient autonomy in decision‐making is constrained by fundamental challenges in accessing NHS care and a limited selection of providers. Patients are not passive consumers of healthcare; they often make decisions based on prior knowledge and their own experiences [8, 32, 33]. However, our research has found that systemic difficulties accessing NHS services discourage patients to seek out comparative information on safety that may deter them from receiving treatment as quickly as possible. For some patients, delaying treatment longer is a safety risk, and so they are reliant on information they already have when selecting a provider to deliver their care.

These findings raise questions about the extent to which patients are able to act on their constitutional rights within the NHS to be involved in decisions about their care. In practice, when there are differences in waiting times between the sectors, combined with the importance patients’ place on quick access, the opportunity for patients to be involved in meaningful and informed decisions may be no more than superficial, and this may constrain patients’ ability to seek out and consider comparative information when making choices. The UK government's ambition to reduce waiting times in NHS hospitals presents a renewed opportunity to strengthen informed decision‐making. Reducing wait times across the system will help to remove the urgency of decision‐making, and provide patients with the opportunity to seek out information and consider all options for their care, as earlier work in this area has called for [34]. However, in the short term, without the implementation of initiatives to engage patients in making choices most aligned with their needs, preferences and priorities, the use of the private sector to reduce waiting lists may do little to improve opportunities for informed decision making if differences in waiting times make one option clearly preferable for many patients.

Our research highlights that although soft intelligence shared through informal relationships can support administrative transitions, it is not a substitute for formal arrangements that guarantee the accurate and timely transfer of patient records between all organisations. This study has demonstrated that more often, disparate systems and inconsistent management of patient records between NHS and private hospitals risk putting the burden for managing care on patients themselves.

Patient accounts of managing their own records have also provided interesting insight into how poor communication between providers undermines patient trust. Where patients recalled past experiences of administrative mismanagement, they took measures to proactively manage their records in later appointments, illustrating how experiential knowledge fosters system knowledge, where patients learn to take an active role in managing their care to more effectively navigate complex healthcare systems [35]. However, participants’ frustration at being asked to repeat aspects of their medical history may also reflect common safety practices in healthcare, where patients are asked to confirm key details to help avoid mistakes or miscommunication. These findings may therefore reflect a gap between the reasons for these checks and how they are understood or experienced by patients.

The erosion of trust by the inconsistent or poor management of patient records also had implications for how safe patients felt when receiving treatment across the interface, regardless of what their care outcomes were. Patient safety literature has expanded the concept of patient safety to encompass patients’ feelings of safety and explores how relational aspects of care between patient and provider foster trust and evoke feelings of safety [36]. Our study contributes to this literature by demonstrating that the visible challenge of informational continuity between public and private providers has implications for patients feeling, and therefore being, safe. Whilst it is evident that organisations must strengthen the systems involved in the transfer of patient information between sectors, they must also consider how to build patient trust in these processes and ensure that the responsibility for managing records does not fall on patients to optimise patient safety in its broadest sense.

Exploring the patient experience of aftercare across the interface between NHS and private providers has highlighted the need to strengthen the management of patient care between sectors, to ensure comprehensive, high‐quality care from beginning to end. Our findings illustrate that the full care pathway is not always clearly defined between NHS and private providers. In instances where complications arose following treatment, unclear arrangements of and responsibility for aftercare between sectors created a diffusion of responsibility that impacted the quality and safety of care that patients received. Our findings extend the understanding of previous research on aftercare in demonstrating that the lack of clarity between NHS and private organisations for the responsibility of aftercare, and resultant communication difficulties, contribute to patients feeling unsafe [37]. Patients expressed conflicting perceptions on who was and who should be responsible for their aftercare, in part determined by how their care was funded. This provides further evidence of the need for organisations to improve their provision of pre‐operative information for what patients can expect at every stage of their care pathway [38] to ensure that aftercare provision aligns with the expectations established when patients choose to receive care across the interface.

### 4.1 Strengths and Limitations

Qualitative interviews with patients and clinical and non‐clinical senior leaders facilitated an in‐depth, holistic exploration of an under‐researched, yet increasingly significant area of healthcare. However, this research focused on the delivery of care in organisations across England, therefore caution should be taken when extrapolating the findings to other contexts. We did not assess participants’ health literacy. It is possible that individuals who volunteered to take part in the study had higher levels of health literacy or particular experiences that motivated them to participate. This may have influenced the themes identified, and findings may not fully represent the perspectives of patients with lower health literacy. It is possible that the sample of patients was skewed towards individuals who wished to offer stronger or more informed responses based on the significance of their experiences than those who did not volunteer to participate. The accounts of patients were also retrospective, from a time when participants were experiencing poor health, which introduces the potential for recall bias. However, the triangulation of data from interviews with senior leaders strengthens the findings from interviews with patients and mitigates the significance of self‐selection and recall bias on the results.

## 5 Conclusion

In exploring the patient experience of elective care across NHS and private sector organisations in England, this study has highlighted the decisions and challenges faced by patients when navigating care across the interface. Despite the increased use of the private sector to deliver patient care in England, there is misalignment between policy expectations and practice in how patients make decisions regarding their care, and inconsistency across organisations in the coordination of care between sectors. NHS and private sector organisations must jointly implement structures that strengthen the arrangements for the delivery of high quality, safe patient care with the inclusion of patients throughout.

## Author Contributions

**Eleanor Gee:** investigation, writing – original draft, writing – review and editing, methodology, visualisation, formal analysis, project administration, data curation. **Gemma Stringer:** investigation, writing – original draft, writing – review and editing, methodology, visualisation, formal analysis, project administration, data curation. **Jane Ferguson:** conceptualisation, investigation, funding acquisition, writing – original draft, writing – review and editing, methodology, visualisation, formal analysis, project administration, data curation. **Michael Molete:** investigation, conceptualisation, funding acquisition, writing – review and editing, methodology, formal analysis. **Kieran Walshe:** conceptualisation, investigation, funding acquisition, writing – review and editing, methodology, visualisation, formal analysis, project administration, supervision. **Christos Grigoroglou:** conceptualisation, funding acquisition, writing – review and editing. **Thomas Allen:** conceptualisation, funding acquisition, writing – review and editing. **Michael Anderson:** writing – review and editing. **Nils Gutacker:** conceptualisation, funding acquisition, writing – review and editing. **Karen Bloor:** conceptualisation, funding acquisition, writing – review and editing.

## Conflicts of Interest

The authors declare no conflicts of interest.

## Supporting information


Supporting File 1

Supporting File 2

Supporting File 3

## Data Availability

The data that support the findings of this study are available from the corresponding author, (G.S.), upon reasonable request.

## References

[1] M. Dayan , S. Gainsbury , and S. Bagri , How Has the Role of the Private Sector Changed in UK Health Care? 2024, 12/11/2025, Available From: https://www.nuffieldtrust.org.uk/news-item/how-has-the-role-of-the-private-sector-changed-in-uk-health-care.

[2] J. Keith , A. Lawless , L. Ewbank , T. Boshari , and T. Gardner , Is the Use of Privately Funded Health Care on the Rise? 2024 [cited 2025 12/11/2025]; Available from: https://www.health.org.uk/reports-and-analysis/analysis/is-the-use-of-privately-funded-health-care-on-the-rise.

[3] DHSC , *Deal Between NHS and Independent Sector to Cut NHS Waiting Lists* (Department of Health and Social Care, 2025).

[4] L. K. Tynkkynen and K. Vrangbæk , “Comparing Public and Private Providers: A Scoping Review of Hospital Services in Europe,” BMC Health Services Research 18, no. 1 (2018): 141. 29482564 10.1186/s12913-018-2953-9 PMC5828324

[5] P. Molander , “Public Versus Private Healthcare Systems in the OECD Area—A Broad Evaluation of Performance,” European Journal of Health Economics 26, no. 7 (2025): 1163–1173. 10.1007/s10198-025-01767-6 PMC12431882 40055294

[6] P. Blomqvist , “Chapter Three: Privatisation and Marketisation Within a Healthcare System: The Swedish Experience,” In Navigating Private and Public Healthcare, ed. F. Collyer and K. Willis (Palgrave Macmillan, 2019), 41–60.

[7] K. Willis and S. Lewis , “Chapter Eleven: The Imperative of Choice in Australian Healthcare,” In Navigating Private and Public Healthcare, ed. F. Collyer and K. Willis (Palgrave Macmillan, 2019), 227–248.

[8] A. Dixon , J. Appleby , R. Robertson , P. Burge , N. Devlin , and H. Magee , How Patients Choose and How Providers Respond (The Kings Fund, 2010).

[9] J. Curtice , C. Sivathasan , and G. Morton , BSA 42| Repairing Britain: Attitudes Towards the Economy, Taxation and Public Services. 2025.

[10] NHS, The NHS Constitution for England. 2023.

[11] NHS. NHS Choice Framework—What Choices Are Available to You in Your NHS Care. 2024, 13/11/2025; Available from: https://www.gov.uk/government/publications/the-nhs-choice-framework/the-nhs-choice-framework-what-choices-are-available-to-me-in-the-nhs#choice-in-the-nhs---an-overview.

[12] Department of Health, Operational Guidance to the NHS: Extending Patient Choice of Provider. 2011.

[13] CMA , *Private Healthcare Market Investigation Order 2014* (Competition and Markets Authority, 2014).

[14] NICE , *Shared Decision Making* (National Institute for Health and Care Excellence, 2021). 34339147

[15] S. S. Radha , N. Caplan , A. St Clair Gibson , M. Shenouda , S. Konan , and D. Kader , “Can Patients Really Make an Informed Choice? An Evaluation of the Availability of Online Information About Consultant Surgeons in the United Kingdom,” BMJ Open 2, no. 4 (2012): e001203. 10.1136/bmjopen-2012-001203 PMC4400617 22918672

[16] C. Exley , N. Rousseau , C. Donaldson , and J. G. Steele , “Beyond Price: Individuals’ Accounts of Deciding to Pay for Private Healthcare Treatment in the UK,” BMC Health Services Research 12, no. 1 (2012): 53. 22397733 10.1186/1472-6963-12-53 PMC3325847

[17] A. Darzi , *Independent Investigation of the National Health Service in England* (Department of Health and Social Care, 2024).

[18] IHPN , Going Private 2024. 2024.

[19] PHIN , *Patient Priorities: Research Into Patient Confidence and Choice in the UK's Private Healthcare Sector* (Private Healthcare Information Network, 2024).

[20] P. Sivey , “The Effect of Waiting Time and Distance on Hospital Choice for English Cataract Patients,” Health Economics 21, no. 4 (2012): 444–456. 21384464 10.1002/hec.1720

[21] J. L. Haggerty , R. J. Reid , G. K. Freeman , B. H. Starfield , C. E. Adair , and R. McKendry , “Continuity of Care: A Multidisciplinary Review,” BMJ 327, no. 7425 (2003): 1219–1221. 14630762 10.1136/bmj.327.7425.1219 PMC274066

[22] G. Freeman and J. Hughes , Continuity of Care and the Patient Experience. 2010, London.

[23] S. Waibel , D. Henao , M. B. Aller , I. Vargas , and M. L. Vazquez , “What Do We Know About Patients’ Perceptions of Continuity of Care? A Meta‐Synthesis of Qualitative Studies,” International Journal for Quality in Health Care 24, no. 1 (2012): 39–48. 22146566 10.1093/intqhc/mzr068

[24] J. Johnson , J. Farnan , P. Barach , et al., “Searching for the Missing Pieces Between the Hospital and Primary Care: Mapping the Patient Process During Care Transitions,” BMJ Quality & Safety 21, no. S1 (2012): i97–i105. 10.1136/bmjqs-2012-001215 23118409

[25] N. Le Maistre , R. Reeves , and A. Coulter , *Patients’ Experience of CHD Choice* (Picker Institute, 2003).

[26] M. Husted , A. Dowrick , R. Porter , et al., “Imposter Participants in Synchronous Qualitative Research: A Systematic Scoping Review,” International Journal of Qualitative Methods 24 (2025): 16094069251342542.

[27] J. M. Roehl and D. J. Harland , “Imposter Participants: Overcoming Methodological Challenges Related to Balancing Participant Privacy With Data Quality When Using Online Recruitment and Data Collection,” Qualitative Report 27, no. 11 (2022): 2469–2485.

[28] J. J. Chandler and G. Paolacci , “Lie for a Dime: When Most Prescreening Responses Are Honest but Most Study Participants Are Impostors,” Social Psychological and Personality Science 8, no. 5 (2017): 500–508.

[29] Y. S. Zhu , A. Wilpers , K. L. Hansen , and J. L. M. McCoyd , “Moving Beyond Red Flags for Imposter Participants: A Novel Screening Strategy to Enhance Data Integrity,” Qualitative Health Research, ahead of print, July 30, 2025, 10.1177/10497323251359207. 40738133

[30] M. Hennink and B. N. Kaiser , “Sample Sizes for Saturation in Qualitative Research: A Systematic Review of Empirical Tests,” Social Science & Medicine (1982) 292 (2022): 114523. 34785096 10.1016/j.socscimed.2021.114523

[31] V. Braun and V. Clarke , “Using Thematic Analysis in Psychology,” Qualitative Research in Psychology 3, no. 2 (2006): 77–101.

[32] P. Dorfman , J. Tritter , M. Koivusalo , and E. Ollila , *Globalisation, Markets and Healthcare Policy: Redrawing the Patient as Consumer* (Routledge, 2009).

[33] J. D. Tariman , D. L. Berry , B. Cochrane , A. Doorenbos , and K. G. Schepp , “Physician, Patient and Contextual Factors Affecting Treatment Decisions in Older Adults With Cancer: A Literature Review,” Oncology Nursing Forum 39, no. 1 (2012): E70–E83. 22201670 10.1188/12.ONF.E70-E83 PMC3247918

[34] A. G. Mulley , C. Trimble , and G. Elwyn , “Stop the Silent Misdiagnosis: Patients’ Preferences Matter,” BMJ 345 (2012): 6572. 10.1136/bmj.e6572 23137819

[35] K. Willis , F. Collyer , S. Lewis , J. Gabe , I. Flaherty , and M. Calnan , “Knowledge Matters: Producing and Using Knowledge to Navigate Healthcare Systems,” Health Sociology Review 25, no. 2 (2016): 202–216.

[36] E. Barrow , R. A. Lear , A. Morbi , et al., “How Do Hospital Inpatients Conceptualise Patient Safety? A Qualitative Interview Study Using Constructivist Grounded Theory,” BMJ Quality & Safety 32, no. 7 (2023): 383–393. 10.1136/bmjqs-2022-014695 36198506

[37] M. Beddard , J. Baker , N. Jones , K. Withers , R. Morris , and J. Torkington , “Qualitative Exploration of Patient and Caregiver Experiences of Bowel Cancer Care in Wales: From Diagnosis to Aftercare,” BMJ Open 15, no. 3 (2025): e088074. 10.1136/bmjopen-2024-088074 PMC11911673 40090680

[38] A. Mottram , “They Are Marvellous With You Whilst You Are in but the Aftercare Is Rubbish’: A Grounded Theory Study of Patients’ and Their Carers’ Experiences After Discharge Following Day Surgery,” Journal of Clinical Nursing 20, no. 21–22 (2011): 3143–3151. 21762418 10.1111/j.1365-2702.2011.03763.x

