# Supplementary material for: Mind the Gap: A Qualitative Study of Patients’ Experiences of Acute Elective Care Across the NHS and the Private Sector in England
Source: Health Expect. 2026 Sep 15;29(5):e70867. doi: 10.1111/hex.70867 (PMC13575767; doi:10.1111/hex.70867)
Supplement: Supplementary file 1 [file HEX-29-e70867-s001.docx]

**Quality, safety and clinical governance in NHS and independent hospitals: lessons from the interface**

**Professional Interview Schedule**

**Before the interview**

1. Welcome and introduction
2. Indicate the time available (e.g. ‘we have around sixty minutes to discuss…’)
3. Repeat consent process (i.e. ask if the participant is happy for the interview to be recorded)
4. Remind participant about confidentiality
5. Set the agenda and take the interviewee through the key areas you will be discussing

*Thank you for agreeing to take part in this project. I’d like to confirm that you have read the participant information sheet and check whether you have any questions about the study? This interview should last approximately one hour. We’d like to remind you that the information collected from interviews will be kept strictly confidential, and your responses will be pseudonymised, so you will not be identifiable in any published data. Any discussions that take place during the study are confidential. However, if you were to tell us something that could put someone at risk of harm or reveal unsafe practice that has not been reported through the usual procedures the researcher might be professionally obliged to report the incident through the normal risk management procedures. Information that indicates harm to patients or professional misconduct will be disclosed by the research team as part of a safeguarding process, in accordance with established good research practice and with the University’s own policy on whistleblowing and public interest disclosure. If this happens, the interview will be stopped and we will discuss with you what we intend to do.*

*The overall aim of this research is to provide evidence on the quality and safety of patient care in NHS and independent hospitals and the effectiveness and impact of shared arrangements for clinical governance. First we’re going to spend a couple of minutes talking about your role in relation to governance and the NHS and independent sector, before moving on to discuss clinical governance relationships between your organisation and other organisations. We’re going to spend the bulk of the interview focussing on [adjust according to individual role]. I’ll ask you about anything you think we’ve missed out of the interview and anything important you think we need to think about. Lastly, we will review what you’ve talked about and have the opportunity to summarise and highlight any key issues you think are particularly important.*

1. Tell us a little bit about your role and how it relates to clinical governance between the NHS and the independent sector.
   a. Who else in your organisation has responsibilities for clinical governance at the interface, what do they do?
2. Which organisations from the NHS/IS do you engage with around clinical governance?
   a. Are there any hospitals where your consultants work that you don’t engage with? Why?
3. Can you tell me about how clinical governance works between your organisation and the NHS/independent hospitals where your consultants work?
4. Thinking about clinical governance, how would you describe the relationships your organisation has with hospitals in the NHS/IS?

Prompts:

- History of relationships
- Impact of the pandemic

When relationships are strong how does this impact clinical governance?

5. How is governance information shared/communicated between sectors?
6. Are there any opportunities for improvement in clinical governance across the sectors?
   a. How do you think these improvements could be implemented?
7. How do you deal with concerns? Can you give me an example.
   a. If there is a concern about a doctor, how is that concern communicated with the NHS/independent hospitals where they work?
   b. When is a concern a concern? What types of concerns are shared? What concerns don’t meet the threshold for sharing?
8. How would you describe the current understanding in the NHS about capacity and capability in the IS?

Prompt: For example in terms of resources, what they can provide and how quality and safety arrangements work?

9. Thinking about appraisal and revalidation – how is information gathered about consultants who work across both sectors?
   a. How is information shared?

**Scope of practice**

10. Thinking about scope of practice – how is information gathered about consultants who work across both sectors?
    a. How is this verified/overseen, can you describe the process? What are the challenges in recording overall scope of practice? How might this be improved?
11. Can you tell me about any shared arrangements with the NHS/independent sector for understanding and overseeing scope of practice?

12. What are the challenges in ensuring doctors in your organisation work within their scope of practice?
    a. Prompt: How do you ensure doctors are only practising in the areas they have appropriate knowledge, skills and experience for?

**Collection and use of consultant performance data**

13. How is consultant performance monitored?
14. How is consultant data collected across the interface?

Prompts:

- How do you share information across organisations?
- How is the data used for identifying possible areas of concern?
- Have there been any challenges collecting consultant performance data?
- How have consultants responded?

**Patient voice**

15. How do you capture patient voice and experiences in relation to the interface between the sectors?

**To wrap up**

We’ve talked about lots of different aspects of quality and safety of patient care in NHS and independent hospitals and the effectiveness and impact of shared arrangements for clinical governance. Rather than me go away with your transcript and decide which are the most important aspects we should focus on, could you summarise what you feel are the most significant aspects we should pay attention to?

Is there anything you’d like to add or anything we’ve not covered?

**Thank you for your time today.**
