# Supplementary material for: Mind the Gap: A Qualitative Study of Patients’ Experiences of Acute Elective Care Across the NHS and the Private Sector in England
Source: Health Expect. 2026 Sep 15;29(5):e70867. doi: 10.1111/hex.70867 (PMC13575767; doi:10.1111/hex.70867)
Supplement: Supplementary file 2 [file HEX-29-e70867-s002.docx]

**Quality, safety and clinical governance in NHS and independent hospitals: lessons from the interface**

**Patient and carer interviews standard operating procedure**

**SOP for interviews**

1. Receive email interest from potential participant.
2. Email exchange with potential participant – send Participant Information Sheet and arrange time for a 5-minute screening call over the phone.
3. Complete screening call using the screening call script [see below]. Take objective notes on the screening process, including participants responses and document the specific inclusion/exclusion criteria that were met or not met. Note any uncertainties, unusual responses or concerns.
a. If successful, send participant the consent form, biographic data form and a Zoom invitation.
b. If unsuccessful, send thank you and reject email.
4. Discuss potential impostor participants with the wider team to make a collective decision on inclusion. Document the team’s decision and rationale.
5. **Ensure that both the consent and bio data forms are received before the interview, send a reminder email to participant if required.** Once received, save both in the relevant folder. Assign a PID and update the interview and biographic data tracker.
6. Complete interview
7. Save the audio file, back-up audio file and interview notes in XX folder.
8. Upload audio file to the transcription service portal. Save in XX folder when the transcription has been completed.
9. Arrange incentive payment and confirm payment timescales with participant.

**Imposter participant red flags and measures**

*Below are potential red flags that suggest an imposter participant (IP) is applying to participate in the research. In cases where an individual is suspected to be an imposter participant, consult with the wider research team before proceeding. An individual should not be automatically rejected if they demonstrate one or two of the flags below.*

| **Recruitment stage** | **Flag** | **Measure** |
| --- | --- | --- |
| Expression of interest over email | -Email content is overly informal, short or follows a similar structure to emails received soon after or before from other individual.  -Email address does not match the name provided or follows a similar format to other email addresses received soon after or before.   -Individual does not have a UK phone number for the screening call OR the individual requests a Zoom call, suggesting that they are not based in the UK. | -Ask the individual to provide their phone number to participate in a screening call. |
| Screening call | -Individual cannot remember the name/s of the hospital/s where they were treated or provide the name of an organisation that does not exist e.g. “Hospital of Oxford”.  -Individual was treated in a hospital, or multiple hospitals, that are not close to where they live and do not provide further detail that explains this.   -Individual is unable or is delayed in response to questions about their treatment or locality.    -Individual cannot remember the name of the patient support group or organisation where they heard about the research opportunity. | Withhold providing detail about the inclusion criteria to ensure that IPs cannot confer to provide responses aligned with the research aims.  Ask the additional questions in the script:   - Where were you treated? *(hospital name)* - Where are you based? |

**Screening call script –** *this is subject to adjustment based on the context of the call and detail that the individual provides.*

“Thank you for joining me on the call, it shouldn’t take more than 5 minutes, I will be asking a couple of questions to check participant eligibility for the study as we do have some restrictions on the participants we are interviewing for this study.

Hopefully you’ve had time to read the Participant Information Sheet, but in summary, we are researching the experience of patients when treated in an NHS and private hospital in England for the same health condition.

- For the purposes of this research, we are just considering physical health conditions, not including maternity or mental health. Please can you confirm that this was a physical health condition*?*
- **If NO -** Unfortunately we are not able to interview you for this study, as we are only interviewing patients with experience of a physical health conditions not including maternity, or mental health. We really do appreciate your interest and thank you for your time on this call. If you have any questions, please email: [research team email address]. [END CALL]
- Where were you treated for your health condition – hospital type (NHS/private)?
- **Follow-up if required:** Were you seen for the same health condition in both the NHS and in a private hospital? *(For example: transferred out of private hospital, private consultation back to NHS for surgery or vice versa).*
- **If NO –** *Were you transferred from a private hospital to an NHS hospital for a health condition?*
  - ***If NO to both -*** Unfortunately we are not able to interview you for this study, as we are only interviewing patients that have been seen in both the NHS and in a private hospital for the same health condition. We really do appreciate your interest and thank you for your time on this call. If you have any questions, please email: [research team email address]. [END CALL]
  - **If YES –** Did you have an overnight stay in either an NHS or private hospital for this health condition or were you treated as a day case procedure?
    **- For a day case:** Was it for a treatment or a procedure/surgery? *Probe if needed to check that it was not just for a test.*

ADDITIONAL Qs:	Where were you treated? *(hospital name)*

Where are you based?

- When were you treated for this health condition?
- For our recruitment records, where did you hear about this opportunity?

We will be in touch shortly to confirm whether we are able to interview you for this research. Do you have any questions before we end the call?

Thank you for your time. If you do have any questions, please email: [researcher email address] and we will be in touch soon. [END CALL]”
