# Supplementary material for: Mind the Gap: A Qualitative Study of Patients’ Experiences of Acute Elective Care Across the NHS and the Private Sector in England
Source: Health Expect. 2026 Sep 15;29(5):e70867. doi: 10.1111/hex.70867 (PMC13575767; doi:10.1111/hex.70867)
Supplement: Supplementary file 3 [file HEX-29-e70867-s003.docx]

**Quality, safety and clinical governance in NHS and independent hospitals: lessons from the interface**

**Patient and carer interview schedule**

**Before the interview**

1. Welcome and introduction
2. Indicate the time available (e.g. ‘we have around sixty minutes to discuss…’)
3. Repeat consent process (i.e. ask if the participant is happy for the interview to be recorded)
4. Remind participant about confidentiality
5. Set the agenda and take the interviewee through the key areas you will be discussing:

*We are carrying out a study to help us understand the quality and safety of patient care in NHS and private hospitals. Patients often move between the NHS and private sector for their care yet we know very little about patient experiences of quality and safety when they are treated in both NHS and private hospitals. We are interested in how the quality and safety of the care provided in NHS and private hospitals differed in terms of your patient journey. I will ask questions such as whether there was an understanding about your history and how the information about your care was shared between the private hospital and the NHS, what happened if you had any concerns or problems and your experience of aftercare and follow-up.*

**Experience of care**

- Could you tell me about your experiences of care in the NHS and private hospitals?
  - What were you treated for and where were you treated?
  - How long did you have to wait for NHS treatment before deciding to access private care?
  - How long did you have to wait for treatment once you decided to access private care?
  - How long ago was this treatment?
- How was your care in the private hospital funded? E.g. NHS funded, funded by yourself, or private medical insurance
  - If privately funded, how do you feel about paying for your healthcare and how do you feel about the affordability of private healthcare?
  - How transparent were the costs involved in accessing private healthcare? Were they any additional unexpected costs?
- How did you come to be treated in that particular hospital?
  - Did you feel you had a choice of where you were treated?
  - If there was a choice, what was important to you in making your choice of hospital? (e.g. distance to hospital, quality of care, affordability)
  - Did you ask anyone for advice on where to seek treatment? (e.g. GP, NHS Consultant, a friend)
  - What other information did you use when deciding which private hospital to seek treatment? (e.g. websites, patient forums, CQC ratings etc)
- What did you know about the private hospital before you went there for treatment?
- What information were you provided with before you went to the hospital?
- Did you know who was responsible for your care when you were in hospital?
- Did you feel fully informed about the risks and benefits of your treatment options was provided by the private hospital? Were you provided information about alternative treatment options before deciding to go ahead with your treatment?
- What did the healthcare staff know about your condition and medical history?
  - What questions did they ask?
  - Did they have access to any information regarding your existing conditions and medical history held by NHS organisations?
- During your stay were you in your own room or on a shared ward? How did this affect your care and how did you feel about your accommodations?
- How did you feel about the care you received? What was the outcome?

**Concerns and safety**

- Did you experience any complications, safety incidents, or near miss events during your treatment in the private hospital?
  - If you did, how were they managed by staff working at the private hospital?
  - Did you feel the staff working at the private hospital were transparent regarding what happened and what interventions have been put in place to avoid this happening again?
- If the patient was transferred, ask the following:
  - Can you tell me why and how you came to be transferred from the private hospital to the NHS hospital?
  - How did you feel about the timing of the transfer?
  - What was your experience of the coordination of services between the NHS and private hospital? For example, were the services relating to your transportation, equipment, medication, and records coordinated/shared?
  - How did the hospitals involve your families in information about transfer?
  - How you feel about this experience? How did it impact you?
- Did you have any concerns regarding your safety during your treatment at the private hospital?
  - If so, were there clear mechanisms to raise these concerns with staff at the private hospital?
  - Once concerns were raised, how were they addressed?
- How safe did you feel in the hospital?
  - What factors contributed to you feeling safe or not safe during your treatment in the private sector?
- What was your experience of care out of hours e.g. evenings and weekends?
- If you have other health conditions, how were these managed by the hospital where you were treated before, and during your treatment? How was your medication management?

**Aftercare**

- What aftercare did you receive?
  - What was your experience of aftercare and follow-up?
  - Was it clear who was responsible for your aftercare?
  - How easy was it to communicate with the private hospital following treatment?
  - How did you feel about your experience of aftercare?
- Overall, how did this experience impact you?

**Anything else?**

Is there anything else you’d like to add or anything that we haven’t covered?

**Sum up**

Based on everything we’ve talked about today what do you feel are the key messages that you want us to think about.
